# Virtual 3D Modeling of Airways in Congenital Heart Defects

**DOI:** 10.3389/fped.2016.00116

**Published:** 2016-10-26

**Authors:** Simone Speggiorin, Saravanan Durairaj, Branko Mimic, Antonio F. Corno

**Affiliations:** ^1^Department of Pediatric and Congenital Cardiac Surgery, East Midlands Congenital Heart Centre, Glenfield Hospital, Leicester, UK; ^2^Department of Pediatric Cardiology, East Midlands Congenital Heart Centre, Glenfield Hospital, Leicester, UK

**Keywords:** airways, 3D cardiac modeling, pediatric cardiac surgery, tracheobronchomalacia, 3D reconstruction

## Abstract

The involvement of the airway is not uncommon in the presence of complex cardiovascular malformations. In these cases, a careful inspection of the relationship between the airway and the vasculature is paramount to plan the surgical procedure. Three-dimensional printing enhanced the visualization of the cardiovascular structure. Unfortunately, IT does not allow to remove selected anatomy to improve the visualization of the surrounding ones. Computerized modeling has the potential to fill this gap by allowing a dynamic handling of different anatomies, increasing the exposure of vessels or bronchi to show their relationship. We started to use this technique to plan the surgical repair in these complex cases where the airway is affected. This technique is routinely used in our Institution as an additional tool in the presurgical assessment. We report four cases in which the airways were compressed by vascular structures – ascending aorta in one, left pulmonary artery sling in one, patent ductus arteriosus in one, and major aorto-pulmonary collateral artery in one. We believe this technique can enhance the understanding of the causes of airway involvement and facilitate the creation of an appropriate surgical plan.

## Introduction

The involvement of the airways is frequently associated with complex congenital cardiovascular malformations, either because of congenital airways malformations (1–3) or because of airways are affected by surrounding vascular structures (4–8).

In these patients, the pre-operative assessment should involve the assessment of the airway as well as the cardiovascular system. This comprehensive approach has shown to produce better clinical results, with reduced complications and costs (9).

In the last few years, 3D printing of heart models has been introduced as an additional tool in a number of pediatric cardiothoracic units, where it has proved very useful in the pre-operative assessment. This new diagnostic modality adds an additional plethora of valuable information about the anatomy (10).

Yet, in the vast majority of cases, the standard clinical practice focuses on the cardiac morphology, while the study of the relationships between cardiovascular structures and airways was limited to conventional investigations such as CT scan or MRI.

The ability to simultaneously assess the two components, cardiovascular structures and airway, and to appreciate their static interactions can increase the understanding of the causes and the extent of pre-operative airway problems and allow anticipation of potential airway issues in the post-operative period. Extrinsic compression of the airways compression can be associated with prolonged post-operative mechanical ventilation, recognized as a strong risk factor for poor outcome after congenital cardiac surgery (8, 11).

The fast move toward 3D printing resulted in underestimating the importance of the computerized modeling (CM). This method allows an assessment of the interaction between the airways and vascular/cardiac structures by selectively removing anatomy from the view. We started using CM in congenital heart patients with airway involvement to evaluate if the application of this technique allows a better understanding of the mutual interactions of the structure inside the chest.

The aim of this study is to demonstrate the potential of this new tool to improve the pre-operative assessment and the surgical treatment of patients with airway problems associated with congenital heart defects.

## Materials and Methods

The patients were studied and treated in the same Institution by the same team.

Each patient underwent a CT angiogram scan using a Seimens Somatom Definition Flash scanner (Seimen Heathcare, Germany). Scan parameters were – non-ECG gated, timed delay, turbo flash, single source, isotropic spatial resolution 0.6 mm, pitch 0.6, and contrast dose of 2 ml/kg with saline follow through. In children, the investigation was done under anesthesia with mechanical ventilation. In the only adult patient, reported in this paper, the investigation was performed under sedation, with patient spontaneously breathing and in expiration.

The datasets were used to create the 3D models using Mimics 18^®^ and 3-matic 10.0^®^ (Materialise NV, Leuven, Belgium).

The segmentation of cardiovascular and airway structures was obtained by using Mimics 18^®^ and following the segmentation process using the blood pool technique as described by Farooqi et al. (12). Once each individual structure was identified and associated with a corresponding mask, the 3D volumetric rendering was obtained. The 3D volume reconstruction was imported into 3-matic for further elaboration. Every single structure was reduced and smoothened to eliminate the artifacts caused by the editing of the dataset. Each single structure was selectively hidden to better visualize the surrounding ones.

The structures of interest were made either transparent or hollow in order to appreciate the effects of the mutual interactions from the intraluminar perspective. The process for each reconstruction took from 1 to 2 h depending on the complexity of the malformation.

Once the final result was obtained, the 3D models of each patient were discussed in a multidisciplinary forum, as routine practice in congenital cardiac surgery.

## Patients

We report four patients where the decision-making process and the surgical plan were guided by a CM evaluation of the anatomy.

### Patient 1

A 3-year-old girl was born with the diagnosis of DiGeorge syndrome, tetralogy of Fallot (TF) with absent pulmonary valve and right aortic arch. The severely dilated pulmonary arteries (PAs) were compressing the airways, and the long-term extrinsic airways compression determined subsequent bronchomalacia with air trapping. TF repair was accomplished with a standard VSD closure, while a Lecompte maneuver (surgical moving of the PAs anterior to the aorta) was performed to relieve the airway compression, after reduction plasty for the dilated PAs. The connection between the right ventricle (RV) and the new PAs confluence was established with a valved conduit. The immediate post-operative period was complicated by prolonged ventilator support, required because of the presence of a significant degree of bronchomalacia, eventually requiring tracheostomy and long-term positive pressure ventilation. At seriate outpatient cardiology follow-up appointments with echocardiography, the RV to PA conduit showed the appearance of progressive stenosis, eventually requiring percutaneous dilatation and stenting. Because of the progressive stenosis at the stent level, surgical upsizing of the RV to PA conduit was indicated.

**Figure 1:**
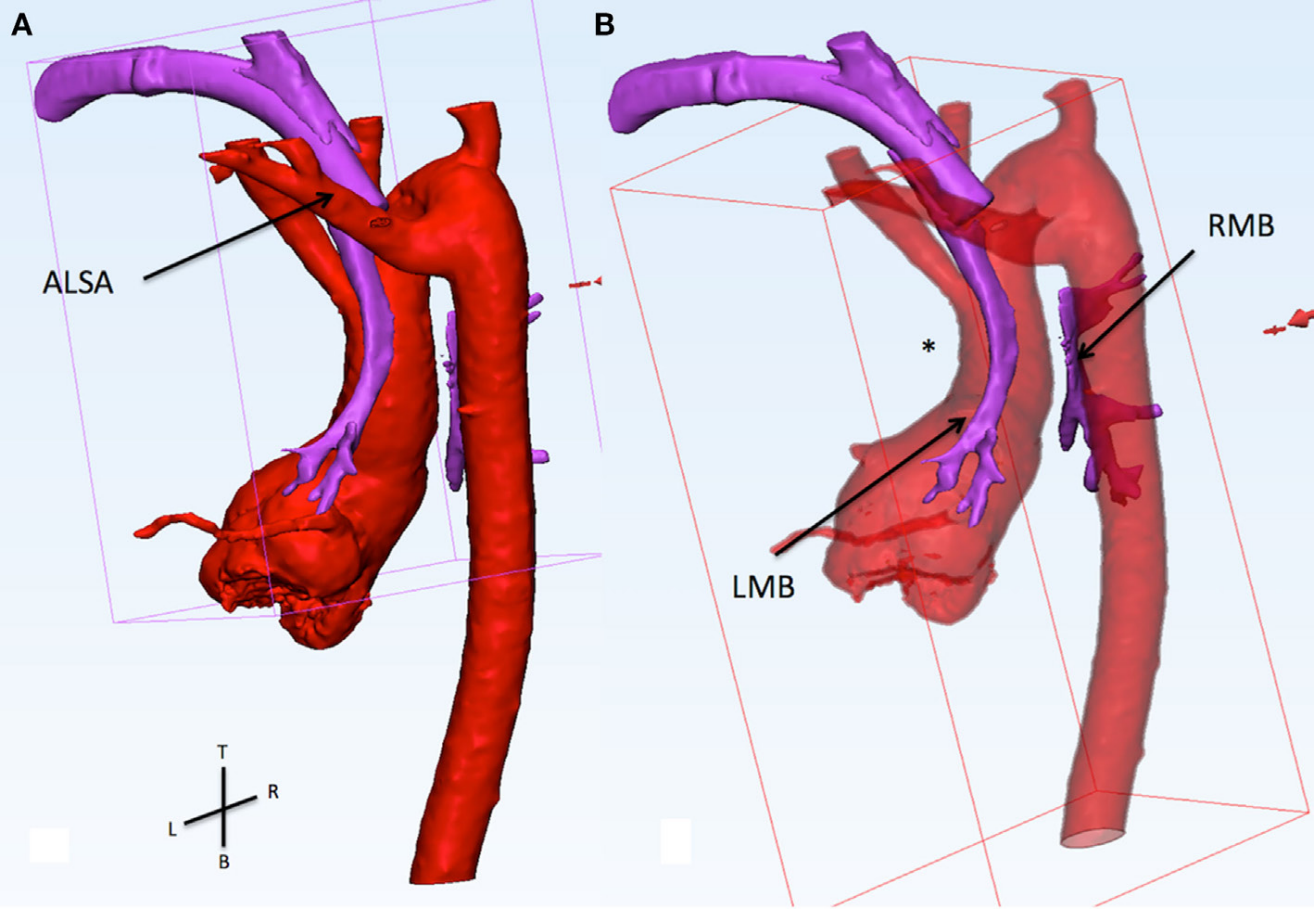
**(A)** This figure shows the relationship of the aorta (red) and the airways (purple). The view is from the back of the patient sand slightly from the left side. The trachea and the tracheostomy tube run between the left carotid artery (anterior) and the aberrant left subclavian artery (ALSA, posterior). **(B)** The ascending aorta presis shown with an abnormal posterior convexity (asterisk) reducing the distance with the descending segment. From the carina, the right main bronchus (RMB) passes between the ascending and descending aorta. In this area, there is a loss of signal of the airways indicating compression. The RMB is wide open distal to the compression.

**Figure 2:**
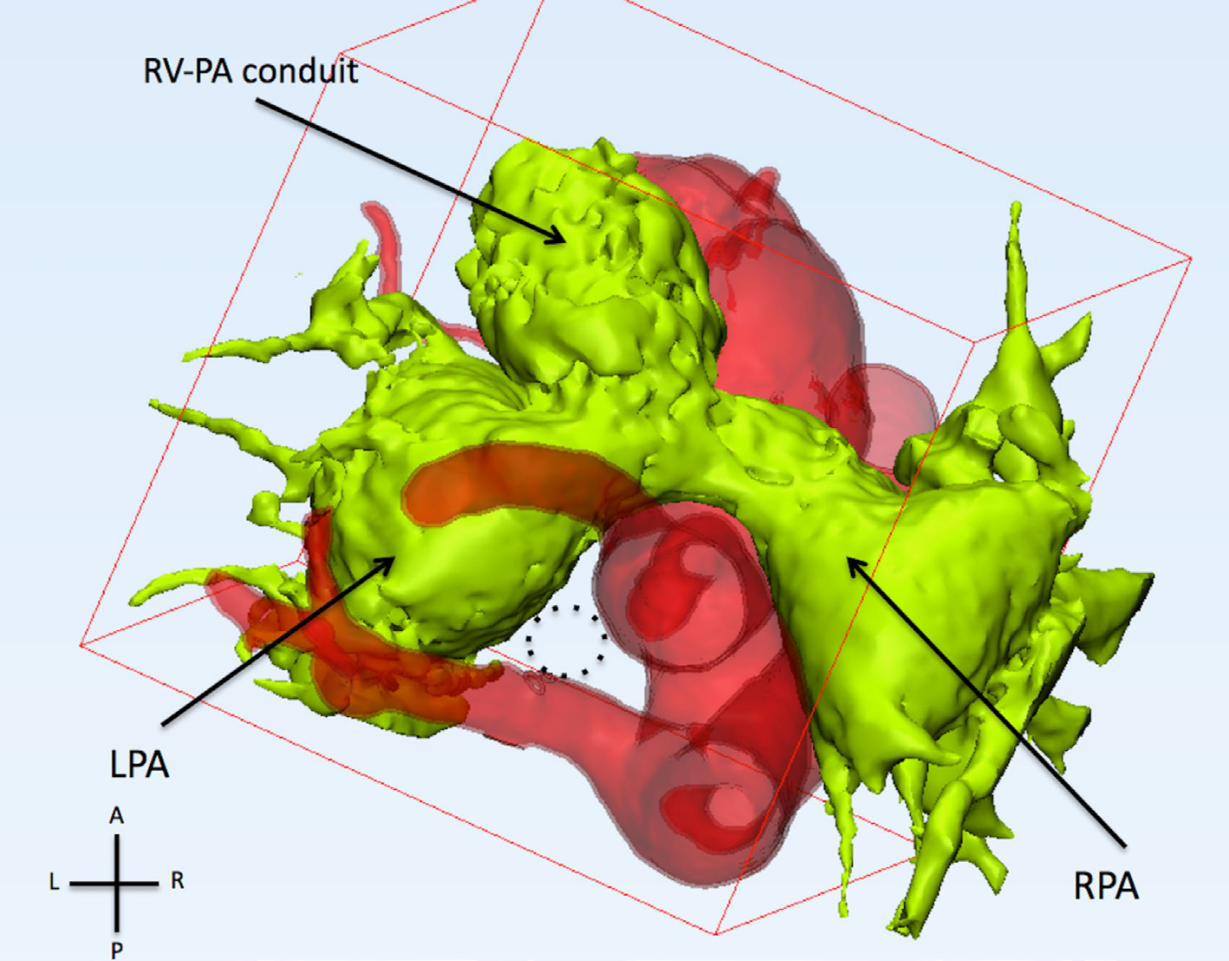
**This figure shows the relationship of the pulmonary arteries and the aorta from the top**. The pulmonary arteries (light green) are anterior to the ascending aorta. The left and right pulmonary arteries (LPA and RPA) are severely dilated. The trachea (dotted circle) runs between the left carotid artery (anterior), aberrant left subclavian artery (posterior), and left pulmonary artery (left).

Computerized modeling showed the distal segment of the trachea compressed between the ascending and descending aorta (Figure 1). The extrinsic compression on the right main bronchus (RMB) was further increased by the fact that the ascending aorta was moved posteriorly by the dilated PA (Figure 2). These finding showed that the only way to relieve the compression on the RMB was to move the ascending aorta away from the bronchus. The posterior convexity of the ascending aorta, appreciated by removing the PAs, reassured the surgical team that there was enough tissue for the aorta to be relocated anterior to the PAs.

After careful repeated median sternotomy, cardiopulmonary bypass was established *via* ascending aorta–bicaval cannulation, on beating heart, the RV–PA conduit was disconnected at its distal connection allowing further dissection. As the pulmonary confluence was transected, the left and right PA sprung apart indicating the tension they had been subjected to. The ascending aorta was carefully dissected from the surrounding scar tissue. Anterograde cardioplegia was administered into the aortic root and aortic clamp positioned. The ascending aorta was transected just above the sinotubular junction, thus visualizing the carina and the origin of the RMB, which were freed from adhesions. The right and left PAs were anastomosed together in front of the carina and posterior to the ascending aorta creating a new confluence. The ascending aorta was anastomosed to the aortic root anterior to the new PAs confluence. Finally, the RV to PA conduit was upsized using an 18-mm Hancock conduit (Medtronic, Minneapolis, MN, USA). The patient was weaned off cardiopulmonary bypass and transferred to pediatric intensive care unit. The immediate post-operative bronchoscopy showed a wider distal trachea with opening of the RMB maintained by lower positive pressure ventilation. The post-operative period was uneventful.

The patient was discharged home on post-operative day 10.

### Patient 2

**Figure 3:**
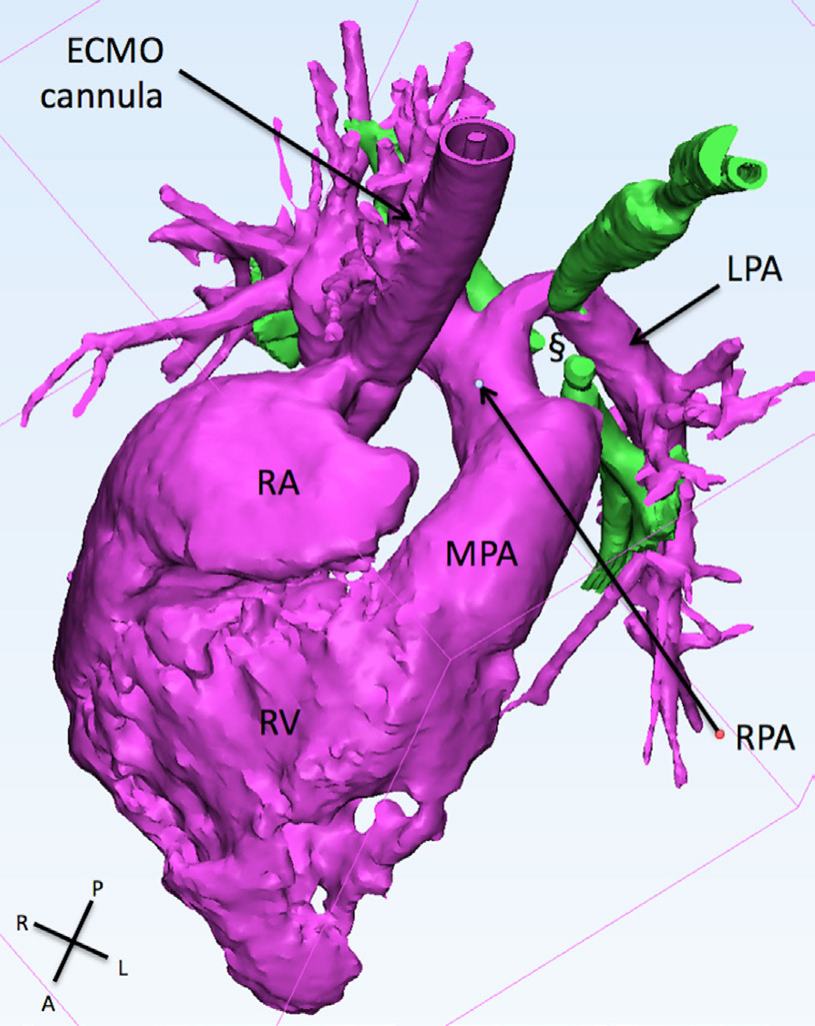
**This figure shows the 3D reconstruction of the right sided structure**. The LPA originates from the right pulmonary artery and slings around the trachea (green) creating a collapse if the latter (§). ECMO, extracorporeal membrane oxygenation; LPA, left pulmonary artery; MPA, main pulmonary artery; RA, right atrium; RPA, right pulmonary artery; RV, right ventricle.

A baby boy was referred to our institution after collapse at birth, rescued, and stabilized by respiratory extra corporeal membrane oxygenation (ECMO). Fiberoptic bronchoscopy showed severe tracheobronchomalacia, mainly affecting the lower third of the trachea and the origin of the left main bronchus (LMB) (Figure 3). After a few days on ECMO, there was no sign of improvement of the lungs, with persistence of severe hyperinflation due to air trapping.

A CT pulmonary angiogram showed the presence of left pulmonary artery (LPA) sling, with severe compression of the lower segment of the trachea and of the origin of both bronchi. The 3D reconstruction showed the absence of any signal at the level of the tracheal carina, with disconnection of both the bronchi from the trachea (Figure 3). The involvement of both the bronchi suggested the presence of an additional mechanism of other than the LPA sling alone. The patent ductus arteriosus (PDA) was found to be the possible additional cause of compression to the RMB. The indication for surgery was then to reimplant the LPA to the MPA and to divide the PDA to open the vascular ring.

*Via* midline sternotomy, on ECMO support, the PDA ligament was doubly ligated and divided. This allowed to remove any tension on tissues at the level of the carina. The LPA was disconnected from the RPA and freed from the tissue behind the trachea. In this way, it was possible to reimplant the LPA to the main PA. The patient was electively kept on ECMO support for 48 h when he was successfully weaning off ECMO support. Orotracheal extubation was achieved on post-operative day 10. The patient was discharged from hospital on 20th post-operative day.

### Patient 3

**Figure 4:**
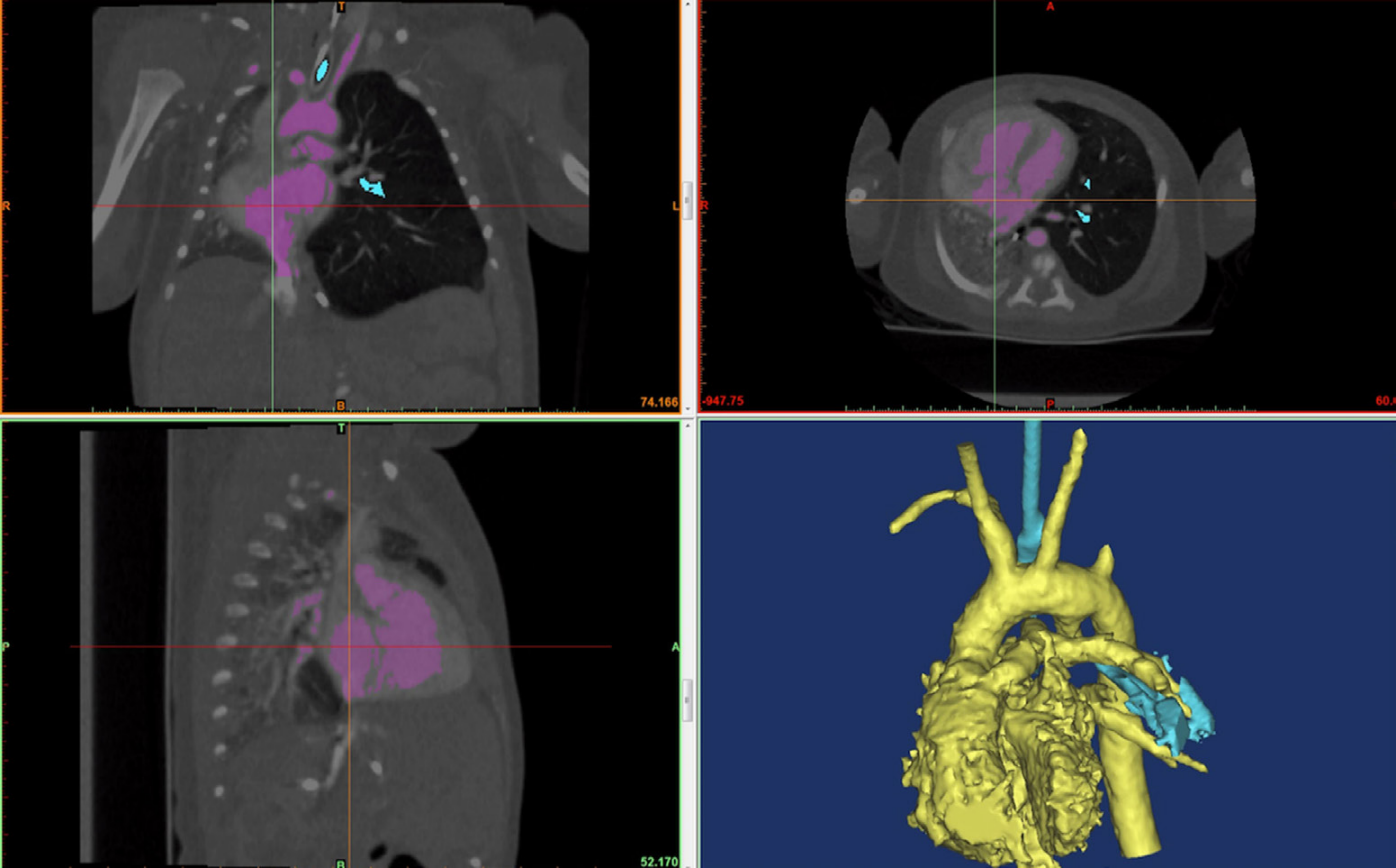
**A screenshot of Mimic^®^ software is shown**. The hyperinflated left lung can be seen in the frontal (top left) and transverse (top right) view. The cardiac mass and the mediastinum are shifted rightwards compressing the left lung.

This is a 4-day-old baby boy with diagnosis of double outlet RV with subaortic VSD, critical PS, malposed great arteries, and tortuous PDA. Non-cardiac abnormalities included dysmorphic features and underdeveloped cerebellar vermis. The chest X-ray showed hyperinflation of the left lung with significant shifting of the cardiac mass into the right chest (Figure 4). Because of the very tortuous appearance of the PDA on the echocardiogram, a CT scan was performed to assess the suitability for PDA stenting.

**Figure 5:**
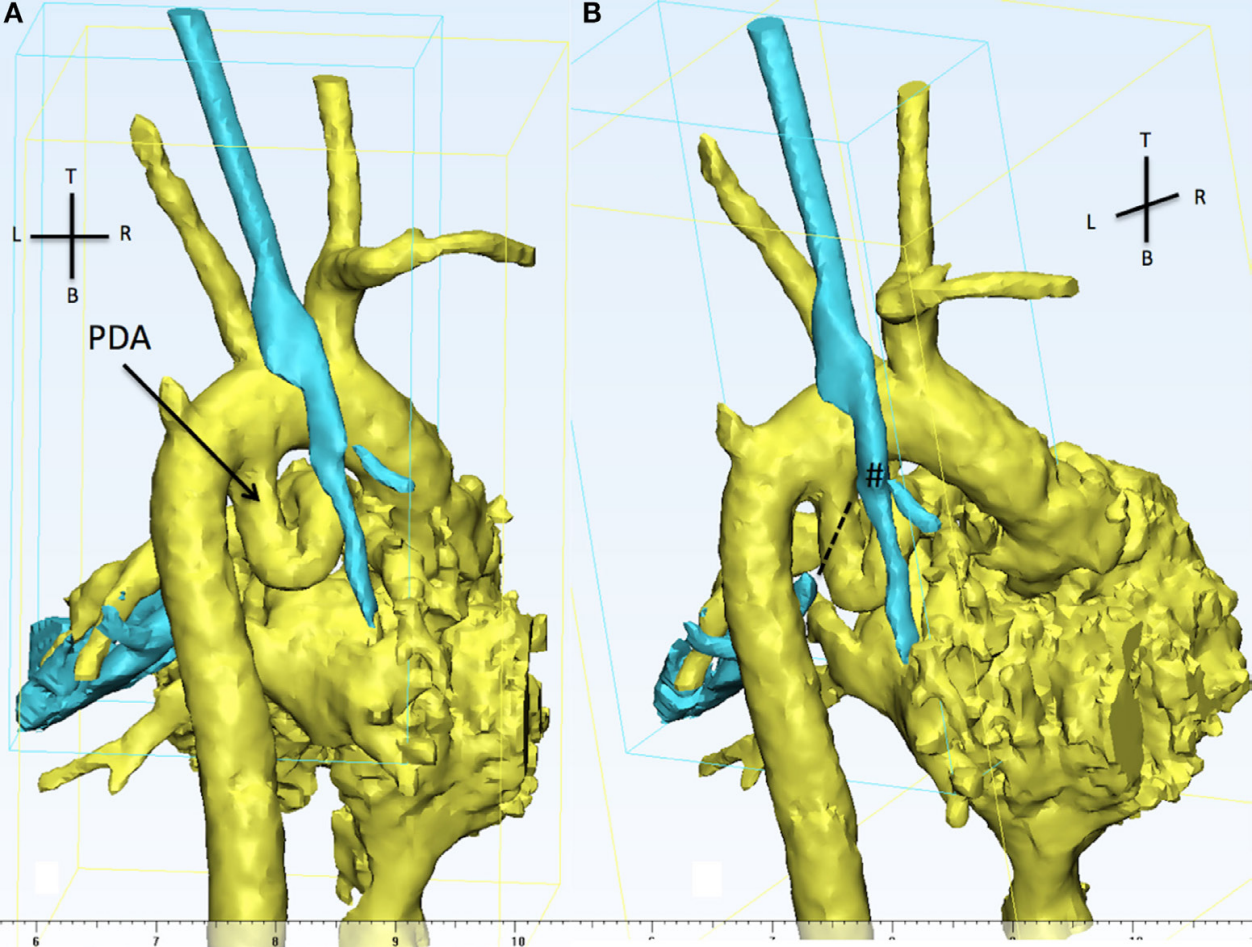
**The 3D reconstruction shows, from the back of the patient, aortic arch, PDA (in yellow), and the trachea (cyan)**. **(A)** The presence of a tortuous PDA taking off from the undersurface of the aortic arch and looping right and upwards. **(B)** The view is still from the back but slightly from the right in order to appreciate the discontinuity between the carina (#) and the distal left main bronchus (dotted line). The LMB is compressed by the descending part of the PDA and the descending aorta. PDA, patent ductus arteriosus.

The 3D reconstruction showed the PDA looping posterior and upwards and then turning anterior toward the main PA. The loop compressed the mid portion of the LMB. The loss of signal of the LMB can be appreciated in Figure 5. Because of the PDA tortuosity, the team felt that stenting was not indicated with limited chances to relieve the compression of the LMB. Indication for high risk surgical PDA division and modified Blalock–Taussig shunt was given. However, the patient was subsequently diagnosed with trisomy 13 and parents provided with comfort care in view of the terminal nature of the condition and hence did not have any intervention.

### Patient 4

This baby boy was born after antenatal diagnosis of pulmonary atresia, VSD, and major aortopulmonary collaterals (MAPCAs). After birth, the pulmonary and systemic circulations were well balanced, maintaining arterial oxygen saturation of 85%.

**Figure 6:**
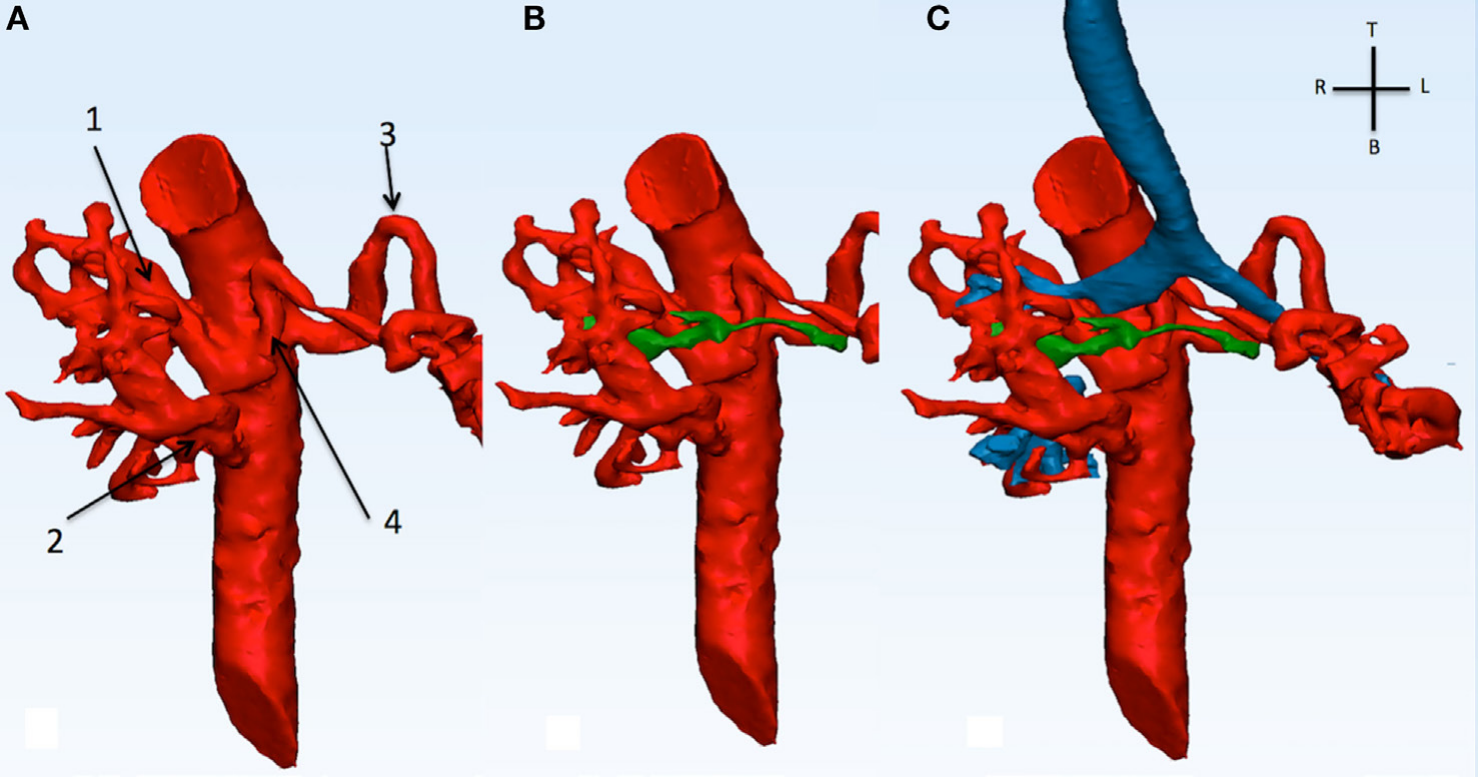
**This picture shows the descending aorta and the origin of the four MAPCAs (A), with the native diminutive pulmonary arteries (green) have been added (B), and with the airways (C)**. MAPCA 1 originates from the anterior surface of the descending aorta with MAPCA 4. MAPCA 1 supplies the right upper lobe, while MAPCA 4 the left upper lobe. MAPCA 2 originates from the distal descending aorta and supplies the middle and lower right lobes. Finally, MAPCA 3 originates from the left lateral aspect of the descending aorta and after a tortuous route it resupplies the left lower lobe. The native pulmonary arteries are shown to be diminutive. The trachea and bronchi can be seen be surrounded by the MAPCAs. MAPCA, major aortopulmonary collateral arteries.

**Figure 7:**
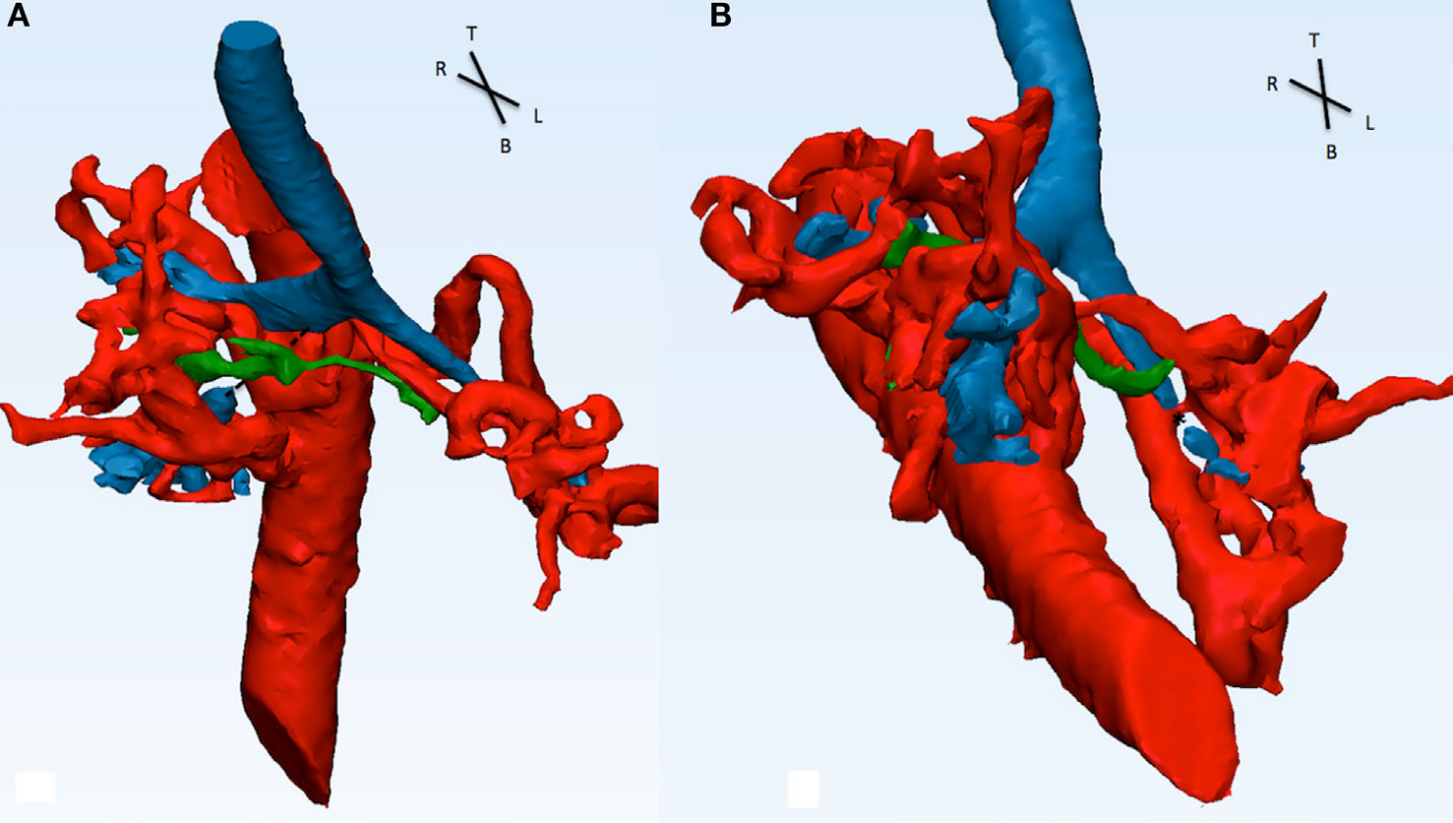
**Descending aorta, MAPCAs (red), native pulmonary arteries (green), and airways (blue) are shown in this picture**. **(A)** From the front, the right intermediate bronchus shows a discontinuity in signal (dotted line) and appears to be compressed by MAPCA 1 (refer to Figure 6) posterior and the native pulmonary artery anterior. **(B)** From the infero-anterior view, the distal part of the LMB is compressed (*) by MAPCA 3 and 4.

A CT angiogram of the thorax and diagnostic cardiac angiogram was performed to evaluate the anatomy of the MAPCAs. The diminutive native PAs are shown in green. The reconstruction shows a lack of signal at the level of the right lower bronchus indicating a vascular compression given by the superior right MAPCA (Figure 6). The distal part of the LMB was compressed by the left sided MAPCA (Figure 7). Because of the compression of the bronchi due to the MAPCAs, the single-stage repair was considered too high risk. A staged approach unifocalization *via* right thoracotomy was chosen to be the appropriate one.

## Discussion

In the last few years, 3D printing has been progressively introduced in an increasing number of institutions substantially improving the way congenital heart malformations are understood by the health carers (i.e., doctors, surgeons, nurses, medical students, etc.) and perceived by parents. The drive behind the acquisition of this new technology was that printed heart models not only allowed for a better understanding of the congenital heart defects but have become extremely useful in the decision-making process as a new tool to define the potential advantages and disadvantages of all the available surgical options. The models are usually a true representation of patient’s heart size.

Unfortunately, the usefulness of the 3D printers has some downsides.

First, the assessment of the cardiac structures and their relationship is a process requiring multiple views from different directions, and sometimes, the structure of interest is not easily exposed because of the surrounding anatomy. Second, high-end 3D printers produce models with a suboptimal and unsatisfactory level of details. This is more evident when dealing with very small structures (e.g., coronary arteries). Finally, in most of the cases, the materials used for the 3D printing cannot be recycled, with negative effects on the environment and elevated costs (13).

The rapid spread of 3D printers moved the focus away from the CM and from the advantages that this technology has compared to printing.

Computerized modeling is a pliable system has the ability to select two of more structures and see their mutual relationship without the interference of the surrounding ones. When modeling, the dataset on the computer, it is possible to zoom, rotate, and identify different structures (or a part of a structure) and remove them or make them transparent in order to appreciate the anatomy of interest. A clear example of these potential is when there is the need to assess the relationship between airway and intrathoracic vessels.

Congenital heart defects can be associated with problems of the tracheobronchial tree (4, 8). In pediatric age, they are generally caused by malacia due to extrinsic vascular compression (5–8), by the presence of complete tracheal rings (1, 3), or occasionally by both (2).

In the presence of extrinsic vascular compression, the assessment of the relationship between vascular/cardiac structures and airways is paramount to understand if and how to relieve the compression.

Because of the central portion that the tracheobronchial tree has in the chest and its close relationship with multiple surrounding vascular structures, it predisposes it to be affected whenever the vessel is dilated or has an abnormal route.

Almost all vascular structures in the chest have been reported to cause airways compression such as the aorta (8, 14), PAs (15), PDA (16), or MAPCAs (17).

In patients with TF and absent pulmonary valve syndrome, the presence of the dilated PAs is a recognized cause of airway compression with subsequent tracheobronchomalacia. The airway symptoms onset may occur very early on in life, particularly because of the associated presence of the right aortic arch (8, 14). A surgical technique to relieve airways compression from dilated PAs was proposed in 2008 by Hraska (15). They proposed the anterior translocation of the PA confluence (Lecompte maneuver) routinely used in arterial switch operations for transposition of the great arteries, following the principle that by moving the PAs away from the airways, there is relief of the airway compression. This technique was adopted in Patient 1, where the anterior translocation of the PAs was performed. Despite this procedure showed promising intermediate-term results in a small group of patients (15), it has also been reported that the PAs can push the aorta posteriorly (with a catapult effect) causing supra-aortic stenosis (18) or persistent airway compression, as in the patient we reported.

Computerized modeling allowed us to separate the various components, such as PA confluence, aorta, and airways, and to appreciate their relationship. By individually assessing each vascular component and at the same time by combining their interactions, it was clear that the origin of the RMB was “pinched” between the ascending and the descending aortas. This compression was aggravated by the tension of the PAs confluence, forcing the ascending aorta to shift posteriorly. We can speculate that the compression caused by the anterior position of the PAs was further increased because of the associated presence of right aortic arch (8).

Some patients with PA, VSD, and MAPCAs can develop airway compromise due to vascular compression by MAPCAs or as a result of the extensive unifocalization (18). MAPCAs can have different anatomical arrangement and very tortuous route. This can lead to airway compression in different sites.

We report a case (Patient 4) where MAPCAs created compression of the right lower bronchus and distal LMB resulting in air trapping of the middle and lower right lung. By using CM, we were able to remove different vascular structures and appreciated which one was contributing the most to the compression and to identify the best approach to relieve the compression.

In complex cases, the ability to independently separate the vascular and airway components (feature that is not available with the routine 3D volume rendering process obtained from the CT scan) allowed the surgical team to identify the precise location and the anatomical cause of airways compression.

Surgery in patients with complex anatomy characterized by multilevel interaction between airways and surrounding vascular structures is extremely challenging and surgeons benefit for as many pre-operative information as possible to have the best understanding of the problem. In some cases, CM can help deciding with best surgical technique; in some others, it may offer an additional level of understanding crucial to plan the surgical repair.

In the case of Patient 1, there are several structural interactions that need to be taken into account. (1) The relationship between the dilated PAs and the aorta: the ascending aorta is forced to have a posterior concavity giving the feel of the tension at the PA confluence level. (2) The relationship between aorta and airways: the distal RMB is disconnected from the carina because of the lack of airway signal on the CT scan. This gap is between the ascending and descending aortas. These two findings supported the surgical plan of “un-doing” the Lecompte.

Computerized modeling allows the user to remove or make transparent the PAs to allow an appreciation of the distorted anatomy of the ascending aorta and its effect on the airways, to isolate the airways and appreciate the length of the bronchial stenosis. This level of detail is not achievable with a 3D printed cast (even if different colors for each individual structure are used!).

## Limitations of the System

Computerized modeling has some limitations. All images are reconstructed from a CT scan dataset and, because of this, they give a static picture of the anatomy without providing additional information of the influence of the respiratory cycle or/and the heart pulsatility. Therefore, at the moment, only speculations can be done about the dynamic interactions between vascular structures and airways.

## Conclusion

The advent of 3D printing allowed pediatric cardiac surgical teams to handle a cast of a heart with a complex or rare malformation before surgery. But it is still very difficult to appreciate interactions between cardiovascular structures and airways.

With CM, it is possible to “deconstruct” the cardiovascular-airway components and precisely identify the problem.

The process we propose not only allows the medical team to appreciate the anatomical relationships but also to evaluate and compare all potential surgical options to relieve any airways compression, thus deciding which approach would be the most effective to improve the patient respiratory status.

## Author Contributions

SS had the idea for the study, performed surgery in two of the patients, wrote the article, collected part of the data, and helped creating the artwork. SD contributed by performing the investigations, collecting databases, drafting, and reviewing the manuscript. BM performed the surgeries, corrected and critiqued the draft of the manuscript, and helped arranging the imaging/artwork. AC had the idea for the study with SS, contributed in modifying and correcting the draft of the manuscript, and the integration with the figures. All authors were actively involved in the creation of this manuscript throughout the period and contributed to its final version.

## Conflict of Interest Statement

The authors declare that the research was conducted in the absence of any commercial or financial relationships that could be construed as a potential conflict of interest.
